# Evaluating the Process and Outcome of a Primary Care Patient and Public Involvement (PPI) Programme: A Pilot Study

**DOI:** 10.1111/hex.70858

**Published:** 2026-08-30

**Authors:** Prawira Oka, Chirk Jenn Ng, Hani Salim, Ping Yein Lee, Ruiheng Ong, Ngiap Chuan Tan

**Affiliations:** ^1^ SingHealth Polyclinics Singapore Singapore; ^2^ Sing Health Duke‐NUS Medical School Family Medicine Academic Clinical Programme Singapore Singapore; ^3^ Centre for Population Health Research and Implementation Singapore; ^4^ Universiti Putra Malaysia Selangor Malaysia; ^5^ Universiti Malaya Kuala Lumpur Malaysia

**Keywords:** family medicine, patient and public involvement, primary care

## Abstract

**Introduction:**

Patient and public involvement (PPI) in research maximises its relevance, quality, and impact by partnering with the public throughout the research journey. In Singapore, a PPI group named Patient Research Advocate and Guidance to Maximise Safety in Trials and Innovation Committee (PRAGMATIC) was established in 2020 by a public primary care institution to create a conducive platform for the public to provide feedback to primary care researchers on their research proposals. This pilot study aimed to evaluate the impact of the committee's feedback on the research process.

**Methods:**

From 2023 to 2024, researchers were invited to present their patient‐fronting research proposals to PRAGMATIC. Before the presentation, researchers submitted a lay‐language research proposal summary that the committee reviewed in advance. The proposals were presented at 3‐monthly PRAGMATIC meetings with the committee providing written and verbal feedback. After each meeting, researchers completed a questionnaire capturing the number, type, acceptability, and helpfulness of the committee's feedback. Reporting followed recommended PPI reporting standards (GRIPP2‐LF).

**Results:**

PRAGMATIC reviewed seven projects and generated 26 potentially actionable feedback. Researchers included two nurses, one medical student, one research associate, and three primary care physicians. Project topics included asthma, postnatal mental health, hearing loss, cervical cancer screening, female smoking, and osteoarthritis. The researchers accepted majority of the feedback (21/26, 80.8%) and rated them as helpful (24/26, 92.3%). The most impact was observed in intervention design and data collection, where feedback led to the adoption of remote interviews, reduced intervention duration, and medical device software refinement. Feedback on participant selection were less accepted but led to improved methodological justification.

**Conclusion:**

Initial evaluations indicate that a structured PPI programme can positively shape study design and implementation in primary care research. These findings are encouraging and have potential to inform future refinements to guide PPI in research.

**Patient or Public Contribution:**

PPI partners were involved in the co‐design of study materials, ensuring their clarity, relevance, and acceptability to participants.

## 1 Introduction

Patient and public involvement (PPI) in research is defined as research that is carried out with or by members of the public rather than to, about, or for them [1]. This movement has grown in recent years with the United States [2], United Kingdom [1], and Canada [3] leading the charge. Organisations such as the US‐based Patient‐Centred Outcomes Research Institute (PCORI) was created in 2009 as part of the Affordable Care Act involving PPI at various stages of the research process [2]. In contrast, awareness regarding PPI in developing nations remains in its infancy [4, 5].

Traditionally, research has been dictated by clinicians, researchers, and the industry with little or no input from the very population it is meant to benefit [1]. By involving the public at various stages of the research journey, the relevance, quality, and impact of the study can be maximised [6, 7]. Despite the inconsistent and underreported impact of PPI on research [7], it has the potential to influence the direction of a study [8], improve subject recruitment [9], and facilitate effective dissemination of study outcomes [10]. Additionally, the benefits of PPI have been widely recognised in the academic space with many reputable journals, such as BMJ, and funders now necessitating it.

However, while the principle of PPI is now widely accepted, the evidence base for how best to structure, support, and evaluate PPI programmes remains limited. Systematic reviews have shown that PPI can have positive effects on research processes and outcomes, but the nature and extent of this impact are heterogeneous and often inconsistently reported [7, 8, 9, 10]. Reviews have also identified numerous frameworks and models for supporting or evaluating PPI, yet there is still limited robust evidence on which programme designs are most effective, for whom, and in what contexts [11].

In Singapore, PPI in health research remains an emerging practice lacking a mandated national framework, with government‐funded research not currently requiring PPI as a condition for funding. Prior local qualitative work suggests individual level PPI faces socio‐cultural barriers in Singapore, including passive citizenship and deference to authority, whereas a community‐based approach may better align with local norms and engagement traditions [12]. Recognising the potential value of PPI in primary care research in Singapore, the Patient Research Advocate and Guidance to Maximise Safety in Trials and Innovation Committee (PRAGMATIC) was established as a context‐specific PPI programme. PRAGMATIC was developed to support the integration of patient and public perspectives into research activities and to improve the relevance and responsiveness of research to the needs of patients, families, and the wider community [12, 13].

Although PPI programmes and panels have been described in other settings, there is limited published evidence on the evaluation of structured PPI models in Asian primary care research contexts [14], particularly those that incorporate formal processes and training for both public contributors and researchers. Evaluating PRAGMATIC therefore offers an opportunity to address this evidence gap by examining the impact of a structured, context‐specific PPI programme on research proposals.

This study aims to evaluate the impact of a structured PRAGMATIC programme on research proposals with specific focus on research objectives, patient selection and recruitment, intervention development, and outcome measures.

## 2 Methods

A pilot study was conducted to determine the impact of PPI on primary care research projects. Reporting followed recommended PPI reporting standards (GRIPP2‐LF) [15]. A completed GRIPP2‐LF checklist for this study is provided in the supplementary materials.

### 2.1 Pragmatic Intervention

PRAGMATIC is a voluntary group of patients and members of public established in 2020 by a public primary care institution to provide a structured platform for the public to provide feedback to primary care researchers on their research proposals. The PRAGMATIC programme aims to: (1) ensure that the community's unique perspectives, concerns and ideas are assimilated into the research activities and (2) collate, prioritise and integrate collective patient perspectives to ensure that research findings are relevant, timely and meet the needs of the person, patients, their families, and the community. The PRAGMATIC programme was hosted by a Family Medicine Academic Clinical Programme, which supports Family Medicine education and research activities.

Under the initial set up, PRAGMATIC met on an ad‐hoc basis every few months to provide input on selected research studies. Due to the COVID‐19 pandemic, these meetings were conducted via teleconferencing. During the session, researchers presented their studies and obtained verbal feedback from the PPI partners. Thereafter, researchers proceeded with their studies, retaining the option to reconsult PRAGMATIC should they encounter challenges during study implementation. Based on feedback from PRAGMATIC and researchers, the PPI review process was revised in 2022 to become more structured and to include training for both PPI partner and researchers. Two PPI partners were involved in refining the workflow, designing the feedback documents, and developing the training programme.

**Figure 1:**
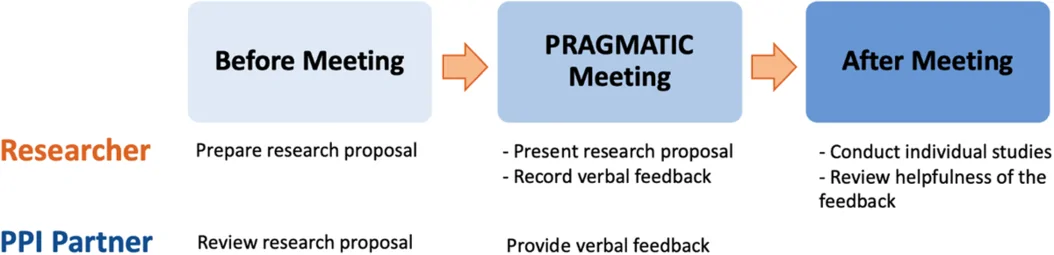
PRAGMATIC intervention.

During the study, PRAGMATIC comprised of 11 adult members (eight females and three males) with varying chronic health conditions. An overview of the PRAGMATIC intervention is depicted in Figure 1.

Between 2023 and 2024, researchers were invited to present their patient‐fronting research proposals at quarterly PRAGMATIC meetings. Two weeks before each meeting, researchers submitted a lay‐language research proposal summary to the committee for their review. During the meeting, researchers presented their proposals and received verbal feedback from PPI partners. Presentations were limited to two per meeting to minimise cognitive overload and encourage active PPI partner participation.

### 2.2 Study Setting and Duration

The study was conducted between 2023 and 2026 at SingHealth Polyclinics (SHP), one of the three public primary care institutions in Singapore. Recruitment of the PPI partners and researchers took place between 2023 and 2024, with 2 years allocated to allow sufficient time for the studies to be completed.

### 2.3 Study Participants

All members of PRAGMATIC were invited to participate in the study. All participants had previously completed a half‐day PPI training workshop that introduced the role of patients, caregivers, and the public as valued partners in research. The workshop covered: (1) the purpose and key principles of PPI; (2) the crucial role of PPI partners as research partners; (3) familiarisation with the PRAGMATIC review process and standardised templates used; (4) opportunities for PPI partner involvement across the research cycle; (5) best practices to foster effective PPI partnerships. Researchers intending to conduct patient‐fronting research at SHP and present their proposals at a PRAGMATIC meeting between 2023 and 2024 were invited to participate in the study via email invitation. Informed consent was obtained from members of PRAGMATIC and eligible researchers.

### 2.4 Research Instruments

This study used three standardised instruments that were co‐developed with PRAGMATIC.

1. Request to PPI Partner for Input on Research Proposal (Appendix 1)
  This lay‐language template included guiding questions to enhance the clarity and understandability for PPI partners.
2. PRAGMATIC Presentation Slide Template
  Researchers utilised this template to present their research proposals. The template incorporated past feedback from PRAGMATIC to improve consistency and encourage meaningful PPI input.
3. PPI Partner Feedback Document (Appendix 2)

The form captured PRAGMATIC input in free‐text, and included options for researchers to evaluate the acceptability (‘accepted’, ‘partially accepted’, or ‘not accepted’) and helpfulness (‘not helpful at all’, ‘not helpful’, ‘somewhat helpful’, ‘helpful’, or ‘very helpful’) of each feedback.

### 2.5 Data Collection

During each meeting, PPI partner feedback were captured by researchers via the standardised PPI Partner Feedback Document. After the meeting, researchers recorded the number and acceptability of the feedback. Following the completion of their studies, researchers evaluated the perceived helpfulness of the PPI input using the same form. Completed PPI partner feedback forms were subsequently collected for analysis.

### 2.6 Data Analysis

The profiles of the researchers were summarised descriptively. Each individual PRAGMATIC input was treated as a separate feedback item and grouped into the following domains: study objective, participant selection, participant recruitment, intervention, data collection, and outcome evaluation. Feedback items were then summarised descriptively according to type, acceptability, and perceived helpfulness.

## 3 Results

A total of eleven PPI partners and seven researchers were recruited into the pilot study (Table 1). PPI partners were mostly female (72.7%) and Chinese (81.8%). Researchers were mostly female (85.7%), with representation across various professional roles. The research topics included chronic diseases, mental health, and preventive care.

**Table 1:** PPI partner (*N* = 11) and researcher demographics (*N* = 7).

PPI partners	*n*
Sex	
Female	8
Male	3
Ethnicity	
Chinese	9
Non‐Chinese	2
*Researchers*	*n*
Sex	
Female	6
Male	1
Professional role	
Doctor	3
Research fellow/officer	2
Medical student	1
Nurse	1
Topic of research	
Asthma	2
Cervical cancer screening	1
Female smoking	1
Hearing loss	1
Osteoarthritis	1
Postnatal mental health	1

**Figure 2:**
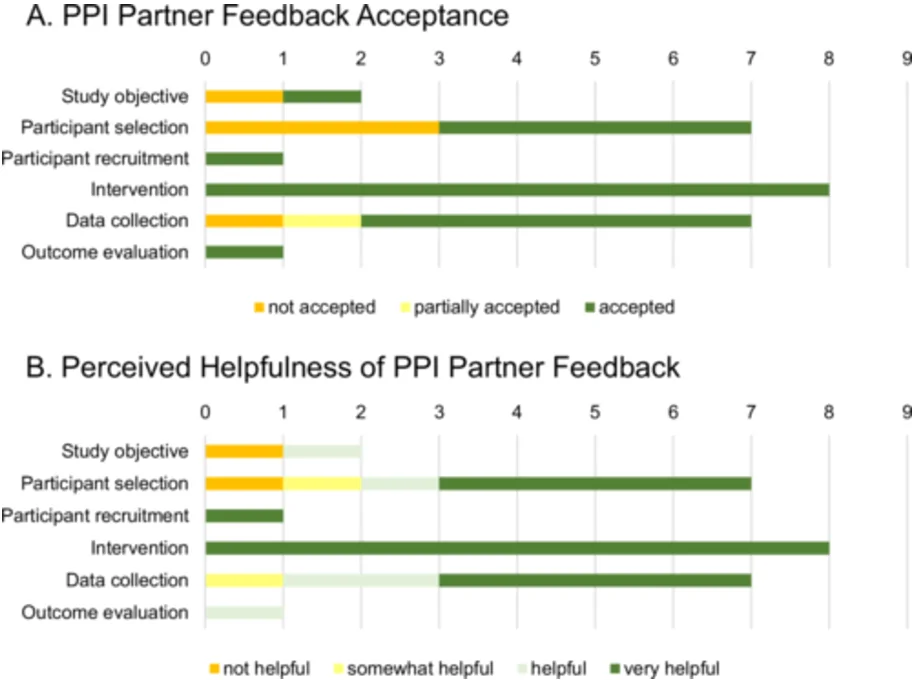
Summary of PPI partner feedback. (A) Distribution of acceptance of PPI partner feedback by researchers. (B) Perceived helpfulness of PPI partner feedback received.

Feedback most commonly related to intervention, participant selection, and data collection, with fewer feedback concerning study objectives, participant recruitment, and outcome evaluation. Figure 2 summarises the 26 distinct feedback provided by PRAGMATIC. Feedback per study ranged from 1 to 6, with a median of 4. The majority were accepted (*n* = 21/26, 80.1%) and found to be helpful (*n* = 24/26, 92.3%). Most feedback were specific to the study intervention design (*n* = 8/26, 30.7%), participant selection (*n* = 7/26, 26.9%), and data collection process (*n* = 7/26, 26.9%). All eight feedback on study intervention was accepted and deemed to be very helpful.

Table 2 provides several examples of the PPI partner feedback that were accepted. All the accepted feedback were deemed helpful and resulted in modifications to the study processes to improve acceptability of study participation.

**Table 2:** PPI partner feedback accepted.

Type of feedback	Example	Comments	Helpfulness
Study objective	‘Be clear about objectives (whether the aim is to increase cervical cancer screening or HPV self‐sampling uptake).’	Nil	Helpful
Participant selection	‘Are you including different age groups? It will be better to include as some may give birth at a later age and their needs may be different from younger mothers.’	‘We included mothers from different age groups to understand any differences in their needs.’	Very helpful
Participant recruitment	‘If a mother has some issues and is having a baby with her, she may be unable to complete the questionnaire or take part in the interview.’	‘A teleconferencing alternative was made available to increase the acceptability of study participation.’	Very helpful
Intervention	‘As a pilot study, don't assign too many activities. Allow the participant to perform them at any time of the day.’	‘Initially, the team wanted to include more activities. The final app design consisted of only 3 activities.’	Very helpful
Data collection	‘Consider individual interviews as participants may have difficulty participating in group interviews due to hearing issues and some may not be comfortable to share in a group setting.’	‘Individual interviews were conducted instead.’	Very helpful
Outcome evaluation	‘How do you measure the research outcome?’	‘The outcomes will include frequency of asthma exacerbations, hospitalisations, and GINA score.’	Helpful

Abbreviations: GINA, Global Initiative for Asthma; HPV, human papillomavirus.

Table 3 summarises the five PPI partner feedback that were not accepted. Despite non‐acceptance, some input were considered helpful as they prompted better justification of study decisions, particularly the participant selection (2 of 3 feedback). In the remaining ‘Data Collection’ feedback deemed to be somewhat helpful, potential delays to the study initiation from the institutional review board (IRB) dissuaded the study team from making changes to the wording of the data collection form.

**Table 3:** PPI partner feedback not accepted.

Type of feedback	Example	Comments	Helpfulness
Study objective	‘Is pregnancy risk a KEY objective? If yes, is it to be part of the research title.’	‘The decision was made not to include pregnancy risk in the title to keep it more well‐rounded and representative of the study's overall scope.’	Not helpful
Participant selection	‘Will you only be including mothers who gave birth 6 months prior? Can it be extended to mothers with older babies, because if it is traumatic, they will probably recall even later. They may be unable to talk about it at the start but could open‐up later.’	‘We identified our target population as mothers who have given birth 6 months or below as that period was identified to be the most stressful and the mothers would be best able to recall any traumatic event due to recency.’	Very helpful
	‘Why are only selected patients included?’	‘The study focused on individuals prone to asthma exacerbations and if the intervention is beneficial to them.’	Somewhat helpful
	‘Why are children not included in the study?’	‘The study site serves mainly adults, thus it was less relevant to focus on children.’	Not helpful
Data collection	‘Improve word choice for the data collection form. Marital status option 5‐ declined to answer, to replace with NA.’	‘Modification would require IRB approval, thus the input was not accepted.’	Somewhat helpful

Abbreviation: IRB, Institutional Review Board.

## 4 Discussion

This study evaluated the impact of a structured PPI programme on key domains of research proposals, namely study objectives, participant selection and recruitment, intervention, data collection, and outcome evaluation. Overall, PPI PRAGMATIC feedback had a positive influence, with most feedback accepted and perceived as helpful. The greatest impact was observed in intervention design and data collection, with most feedback being implemented to enhance feasibility and participant acceptability. Crucially, even non‐accepted feedback led to strengthened methodological justification through improved transparency and clarity of study decisions.

### 4.1 PPI Feedback Accepted

PPI input had the most impact on intervention design, with all feedback accepted and rated as helpful. The findings indicate strong alignment between PPI input and researchers' priorities in developing feasible participant‐centred interventions. Feedback focused on improving participant experience through minimising inconvenience, complexity, and discomfort of study procedures. PPI feedback across the reviewed proposals (Table 2), for instance to reduce the frequency of study visits and duration of wearable device usage, highlight practical considerations that may potentially be overlooked by the study team. Incorporating such inputs early, at the proposal stage, allows researchers to optimise methodological rigour and participant acceptability, which has been linked with improved study participation [11]. Similarly, PPI partner's inputs input resulted in modifications to the data collection process. As reported in the results, one PPI partner highlighted that participants with hearing impairment may experience difficulties participating in group interviews or feel uncomfortable doing so. In response, individual in‐depth interviews were conducted instead of focus group discussions, allowing participants to share their experiences in a more private and conducive setting. Such examples highlight the value of PPI in qualitative studies [16], where patient comfort is associated with richer data capture [17].

### 4.2 PPI Feedback Not Accepted

In contrast, most PPI input that were not accepted were related to participant selection, specifically on broadening the eligibility criteria. While these feedback were generally not accepted, they prompted the researcher to improve the clarity and justification of the target population. This underscores the potential role of PPI partners in challenging assumptions and strengthening methodological rigour even when feedback are not adopted [6]. Aside from methodological considerations, logistical limitations pertaining to anticipated delays from the ethics approval process precluded the acceptance of one feedback. Although the impact in this study was likely minimal, it highlights the practical constraints of incorporating PPI input in research after ethics approval has been granted. While the UK National Institute for Health and Care Research (NIHR) recommends incorporating PPI early in the study design phase [18], its practical integration may still be impeded by timelines required for ethical approval and study commencement. Once ethics approval is obtained, the protocol becomes fixed, with even subsequent minor post‐approval changes likely necessitating formal ethics board re‐review, thereby limiting the incorporation of modifications perceived to be minor by the study team. Therefore, it is crucial to establish clear and reasonable timelines for PPI in research to avoid tokenistic engagement and maximise its impact [19].

### 4.3 Limitations and Strengths

In this study, impact was assessed through researcher‐reported acceptance and perceived helpfulness of feedback, rather than objective downstream measures such as study implementations or outcomes. Additionally, assessment of usefulness did not incorporate objective measures and was instead subjective and contextual to each research team and stage of the research cycle. Future larger comparative studies should be performed to evaluate the impact of PPI on research across different stages of the research cycle incorporating participant recruitment, retention, and outcomes. Moreover, this was a small pilot study involving seven projects within a single institution, so the findings should be interpreted as preliminary and may have limited transferability to other settings.

Despite these limitations, this study has several strengths. To the best of our knowledge, it is among the first in the Asia‐Pacific region to utilise the GRIPP2‐LF to report the impact of PPI input on research proposals in the primary care setting. Assessing both the acceptance and helpfulness of feedback facilitated a more holistic evaluation of PPI impact, beyond simply reporting whether feedback was incorporated. Furthermore, the research proposals spanned diverse topics, capturing the range of contexts where PPI has the potential to make an impact.

## 5 Conclusion

Initial evaluations indicate that a structured PPI programme can positively shape study design and implementation in primary care research. These findings, while preliminary given the small sample size, are encouraging and highlight the practical value of engaging PPI partners early. Future work involving a larger number of proposals would help confirm whether these benefits are generalisable. Nonetheless, this work adds to the growing body of evidence on PPI programmes and has the potential to inform future refinements to guide PPI in research.

## Author Contributions

**Prawira Oka:** conceptualisation, investigation, writing – original draft, methodology, writing – review and editing, formal analysis, project administration, data curation, visualisation. **Chirk Jenn Ng:** conceptualisation, writing – review and editing, visualisation, methodology, supervision, formal analysis, writing – original draft. **Hani Salim:** conceptualisation, methodology, writing – review and editing, supervision. **Ping Yein Lee:** conceptualisation, methodology, writing – review and editing, supervision. **Ruiheng Ong:** writing – review and editing, investigation, conceptualisation. **Ngiap Chuan Tan:** supervision, resources, writing – review and editing, funding acquisition, conceptualisation, methodology, investigation.

## Ethics Statment

The study was approved by the SingHealth centralised institutional review board (CIRB no: 2023/2128) on 26 April 2023. Informed consent was obtained from participants before study involvement.

## Conflicts of Interest

The authors declare no conflicts of interest.

## Data Availability

The data that support the findings of this study are available on request from the corresponding author. The data are not publicly available due to privacy or ethical restrictions.
